# Wearable IMU-Based Human Activity Recognition Algorithm for Clinical Balance Assessment Using 1D-CNN and GRU Ensemble Model

**DOI:** 10.3390/s21227628

**Published:** 2021-11-17

**Authors:** Yeon-Wook Kim, Kyung-Lim Joa, Han-Young Jeong, Sangmin Lee

**Affiliations:** 1 Department of Smart Engineering Program in Biomedical Science & Engineering, Inha University, Incheon 22212, Korea; kimywih1@naver.com; 2 Department of Physical and Rehabilitation Medicine, Inha University Hospital, Incheon 22332, Korea; drjoakl@gmail.com (K.-L.J.); rmjung@inha.ac.kr (H.-Y.J.); 3 Department of Electronic Engineering, Inha University, Incheon 22212, Korea

**Keywords:** balance assessment, data augmentation, gated recurrent unit, human activity recognition, inertial measurement unit, one-dimensional convolutional neural network

## Abstract

In this study, a wearable inertial measurement unit system was introduced to assess patients via the Berg balance scale (BBS), a clinical test for balance assessment. For this purpose, an automatic scoring algorithm was developed. The principal aim of this study is to improve the performance of the machine-learning-based method by introducing a deep-learning algorithm. A one-dimensional (1D) convolutional neural network (CNN) and a gated recurrent unit (GRU) that shows good performance in multivariate time-series data were used as model components to find the optimal ensemble model. Various structures were tested, and a stacking ensemble model with a simple meta-learner after two 1D-CNN heads and one GRU head showed the best performance. Additionally, model performance was enhanced by improving the dataset via preprocessing. The data were down sampled, an appropriate sampling rate was found, and the training and evaluation times of the model were improved. Using an augmentation process, the data imbalance problem was solved, and model accuracy was improved. The maximum accuracy of 14 BBS tasks using the model was 98.4%, which is superior to the results of previous studies.

## 1. Introduction

Elderly, brain-damaged, and rehabilitation patients often have poor balance. If this and related conditions are not diagnosed promptly, the patients are more likely to suffer further injury by falling [1,2]. Recently, human activity recognition (HAR) was introduced to monitor the motion of a subject in daily life using healthcare devices to determine measures to prevent such accidents [3,4,5].

In HAR research, various sensors are used, such as an inertial measurement unit (IMU), vision sensors, electrocardiograms (ECGs), and electromyography (EMG) devices [6]. For example, a recent HAR study used a textile stretch sensor attached to patients‘ clothing [7]. The IMU-based HAR is among the most popular research targets. Even if a non-invasive method is used, such as EMG and ECG, the connections are often unreliable, and they must be changed often, creating fallacious artifact signals [8]. Although cameras are an option, there are limitations to camera installation, owing to bulkiness and obstruction, not to mention privacy issues. Furthermore, lighting and spacing are often problematic [9]. On the other hand, IMUs avoid these problems. Microelectromechanical IMU systems have small size, low cost, and low operational power requirements. Hence, they can be implemented as wearable devices (e.g., smartwatches, fitness bands, and smart clothing [9,10]). Because human health problems are most often expressed as measurable behaviors [11], IMUs are more suitable for daily activity data collection than other sensors. Hence, many IMU-based HAR studies have been accomplished [3,4,5,9]. Digo’s study [12] was conducted to effectively recognize the working condition by wearing only one IMU in the trunk position. This may allow users to wear fewer IMUs, making it easier to use IMUs in daily life. Studies on user motion recognition using an IMU in a smartphone were also performed to recognize motion for daily life [13,14,15].

Machine-learning models have been widely used [16] for sensor-based HAR measurement, allowing manual and heuristic features to be extracted. Jianchao’s study [17] attempted to classify daily patient behaviors with an IMU using global and local features. Global features (e.g., mean and variance) are typically captured using sliding-window techniques, whereas local features typically contain correlation information, such as similarity and error rates. A final feature vector can be determined by using a feature selection algorithm, which shows better classification performance than other methods. In studies using machine learning, the feature selection method often determines the model’s performance. In order to extract good manual and heuristic features, sufficient understanding of data and signal processing algorithms is required. Hence, it takes a great deal of time and effort to obtain the desired results because selections and combinations of features must be manually verified [18]. By comparison, deep learning uses raw data as input, and all feature extraction and classification procedures are mathematically combined automatically. Therefore, the process is not only fast and convenient, but it also has the advantage that human error is far less likely. Even features that cannot be recognized by humans can be mathematically extracted. Hence, recently, deep-learning algorithms have been employed more often than machine learning methods, and they have performed well [19]. In a paper by Nathanial Pickle [20], an algorithm was used to estimate whole-body angular momentum and directly determined imbalances by learning wearable IMU data with a one-layer artificial neural network, achieving good performance. In Chung’s HAR study [21], data from a multimodal nine degrees-of-freedom IMU was used, and an ensemble model comprising a long short-term memory (LSTM) for each head modality was proposed. Among the many deep-learning studies, the mixed-model convolutional neural network (CNN)/recurrent neural network (RNN) showed better performance than machine-learning models, CNNs, and RNNs alone in many studies [22]. Mekruksa Vanich et al. [23] studied an HAR algorithm using a built-in smartphone IMU. They proposed a four-layer CNN–LSTM model that outperformed the stand-alone LSTM machine-learning model. Mekruksa-Vanich’s study [24] demonstrated a CNN-bidirectional gated recurrent unit (GRU) model that showed better classification performance than a machine-learning model, including a CNN with a GRU, against several IMU-based HAR open datasets.

In this work, an HAR algorithm is examined by introducing an IMU system to assess patients via the Berg balance scale (BBS), a clinical test for balance assessment. The BBS is a highly reliable balance test for elderly and stroke patients [25,26]. It consists of 14 static and dynamic motion tasks performed in daily life. Each motion is scored, and the total score is used to assess the patient’s probability of falling. A previous study [27] used a machine-learning model for the scoring algorithm. Sensor data with high-scoring contributions were selected for each task. The sum of the energy in the front and rear sections of the motion data was used as a feature, and the amplitudes of frequency components up to 15 Hz were also used. The features were selected by kernel principal component analysis (a feature extraction algorithm), and the data were classified using a support vector machine (SVM). The results showed excellent performance, which improved the performance of Badura’s study [3]. However, because these studies used machine-learning models, a great deal of time and effort was required to extract and verify the manual and heuristic features. Hence, in this study, the final feature vector is extracted and classified using a deep-learning algorithm, thus reducing the time and effort requirements of the feature extraction process. It also improves the scoring accuracy. Furthermore, the dataset of the previous study is improved using a signal-processing algorithm, and the performance and computational efficiency of the model are improved. The dataset of the previous paper had a data imbalance problem. To resolve this, an oversampling-based data augmentation process was performed to equalize the amount of data for each class and to increase the total amount of data. These efforts led to an increase in the accuracy of the model. Additionally, the previous data suffered oversampling of participants’ movement data. This was resolved by finding an optimal sampling rate of data. The sampling rate of the data was reduced. Hence, it is now possible to reduce the computational complexity while preserving classification accuracy. This also lowers the data-sampling rate of the IMU module, which contributes to reducing the power consumption of the wearable device. Furthermore, we further improve the deep-learning model by optimizing the GRU and one-dimensional (1D) CNN models with a shallow structure in consideration of our small dataset. Additionally, performance improvement was achieved by using an ensemble of the two models. As with previous papers, we attempted to create a single model that can cover all BBS tasks. As a result of our experiments, the model comprising two 1D-CNN heads and one GRU head stacking ensemble model had the highest average accuracy on all 14 tasks. This result was superior to previous results [3,27].

## 2. Materials and Methods

### 2.1. Experiment

#### 2.1.1. Motion and Experimental Protocol

The BBS was devised to assess the balance of elderly and stroke patients [25,26]. For this exam, subjects are asked to perform 14 functional tasks, and a rehabilitation therapist assigns a score from 0 to 4 for each task. Combined scores of 0 to 20, 21 to 40, and 41 to 56 represent balance impairment, acceptable balance, and good balance, respectively. Table 1 presents a description of the 14 BBS tasks.

The experiment was performed at the Stroke (brain injury) rehabilitation clinic of the Department of Rehabilitation, Inha University Hospital. The patient wore a wearable IMUs and performed BBS with a rehabilitation therapist in the same manner as the usual BBS assessment. Some patients could not do all tasks, and they perform only tasks that they could do. Patient data had a data imbalance problem in which the amount of data for each score was different and some scores had no data. Therefore, a healthy participant experiment was conducted to complement the lack of data. Healthy participant experiments were advised by rehabilitation specialists and experimented under the coach of rehabilitation therapists. The healthy participants conducted experiments that imitated the patient’s movements. The healthy participants performed all the motions with a score of 0 to 4 in the 14 tasks of the BBS assessment. Therefore, in the experiment with healthy participants, it was possible to obtain the same as five times of experiment data per person.

#### 2.1.2. Participants

**Figure 1:**
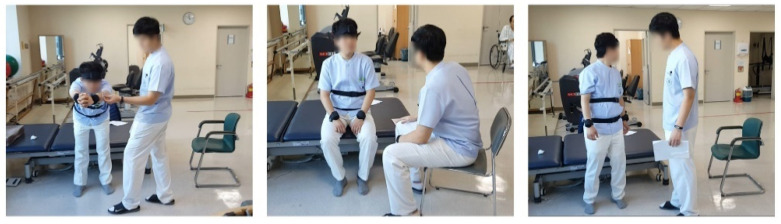
Scenes of BBS experiment conducted with a healthy participant.

This study was approved by the Institutional Review Board of Inha University Hospital. Among hospitalized brain disease patients, those expected to be at risk of falling due to poor balance participated. The diseases of each patient differed slightly, but each had either cerebral infarction, cerebral hemorrhage, brain atrophy, or brain embolism. A total of 53 patients (31 male and 22 female) participated, and their ages ranged from 50 to 80 years. The mean age was 64.9, and the standard deviation was 12.6. The healthy experimental participants included three males in their 20s. The average age of healthy participants was 28.7, and the standard deviation of age was 0.6. Figure 1 shows scenes of the BBS experiment conducted with a healthy participant.

#### 2.1.3. Equipment and Data

**Figure 2:**
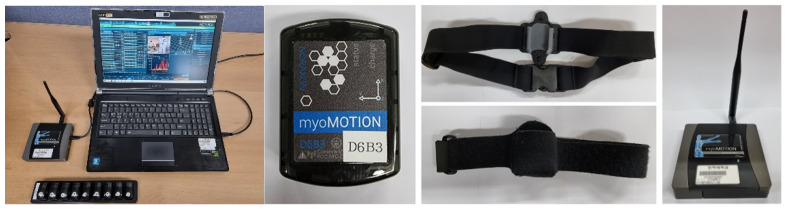
Configuration of the Noraxon myoMotion.

Noraxon’s myoMotion was used for the experiment. This equipment is a multichannel wireless IMU system certified as an ISO 13485 compliant (Registration # MED-0037b) and an FDA 510 K compliant (Registration number #2098416) medical device. The system consists of a multi-channel IMU module capable of wireless data transmission, a receiver, and a Velcro band for attaching the IMU to the human body. The receiver was connected to a computer via USB and records the received data using the provided software. By adding a USB webcam to the configuration, it can record video time-synchronized with IMU data. Because the recorded video and IMU data can be checked simultaneously, the video can be used as the golden state of the IMU data. Figure 2 shows the configuration of the Noraxon myoMotion.

**Figure 3:**
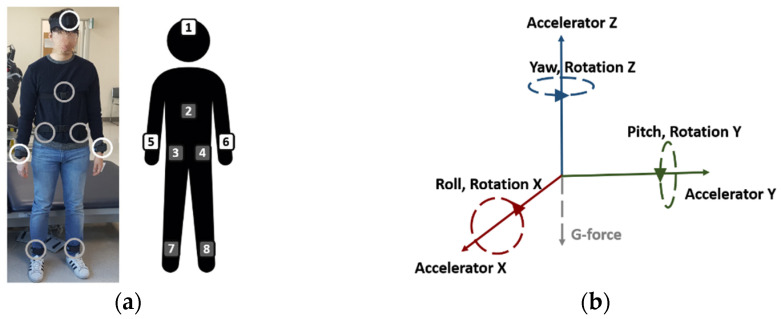
(**a**) Position of wearable IMU sensor; (**b**) the types of IMU sensor data.

IMU sensors were attached at eight locations: the forehead (FH), back (B), both wrists (RtW: right wrist, LtW: left wrist), both ankles (RtA: right ankle, LtA: left ankle), and both hips (right and left hips). Each IMU sensor yielded nine types of sensor data: three-dimensional (3D) acceleration data ($Ac_{x},Ac_{y},Ac_{z}$); and data that excluded gravity and pitch (P), roll (R), yaw (Y), and 3D rotation data ($Ro_{x},Ro_{y},Ro_{z})$. The rotation data contained the number of accumulated rotations for each 3D axis. The sampling rate of the data was 100 Hz. Figure 3 shows the position of wearable IMU and the types of IMU sensor data.

#### 2.1.4. Output Data Description

Each IMU outputs nine data types, owing to the eight IMUs used. This provides a total of 72-dimensional time series data items output in real time. Additionally, video was recorded for use as a golden state of IMU data. The duration of the experiment for patient participants was approximately 10–15 min. Some patients could not perform all 14 tasks, resulting in shorter performance times. Fifty-three experimental data were recorded from the patients. Three healthy participants performed all the motions from score 0 to score 4, and 15 experimental data were obtained from the healthy participants. Therefore, the equivalent of 78 experimental data were recorded.

### 2.2. Methodology of the Proposed Method

**Figure 4:**
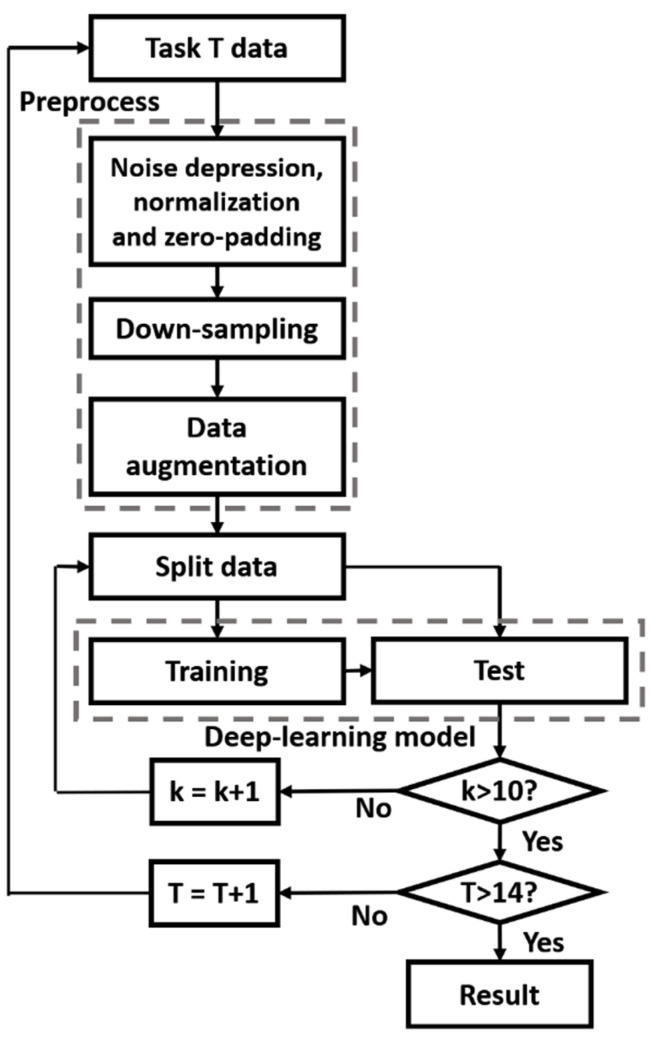
Methodology of the proposed method.

Figure 4 presents the methodology of the proposed method. Before training the deep-learning model with the data, the dataset was improved, and 14 models were evaluated by 10-fold cross-validation.

#### 2.2.1. Preprocess

##### Noise Depression, Normalization, and Zero-Padding

The high-frequency noise of an inertial sensor can be removed using an empirical mode decomposition algorithm [28]. As in a previous study [27], the signal was decomposed into 10 intrinsic mode function (IMF) components and resynthesized from the first to the seventh IMF (low-frequency components) to remove the high-frequency component considered to be noise. Min–max feature scaling normalization was performed on the data to prevent the model from being biased to large values caused by unit differences. Multivariate time-series motion data output from the eight wearable IMUs were continuously recorded from the start to the end of the assessment. Therefore, it was necessary to extract only the section in which the assessment was performed from the data. Using the video, each task execution section was identified, and the IMU data of this section was used as task data. The data length for each task was set to the longest data item in the task, and zero paddings were performed at the end of the data having short lengths.

##### Data Down-Sampling

According to a previous IMU-based HAR study, even when the sampling rate for 100–250 Hz data was decreased to 12–42 Hz [29], the performance reduction was small, and even the sampling rate of 10 Hz was sufficiently recognizable [21]. Likewise, because the sampling rate of the BBS motion data was 100 Hz, adequate down-sampling was expected to improve the efficiency of the model. To select an appropriate sampling rate, the accumulated information with respect to the frequency was observed, and the accuracy, training time, and evaluation time before and after down-sampling were compared with a classification model. The process is described in detail in Steps 1–5.

1. From the first person in Task 1, an n-point fixed Fourier transform was applied to each of the 72 sensor data outputs from the eight IMUs, and amplitudes from first to the n/2th were extracted.
2. For each person, the amplitude values of all sensors were summed for each frequency component. The accumulated amplitude value for each N Hz frequency was calculated, where N = {1, 2, 3…50}. The accumulated amplitude for each frequency was divided by the sum of the amplitudes up to 50 Hz, which is the sum of all frequency components, and multiplied by 100 to obtain the percentage (%). Thereafter, the average percentage of the accumulated data for each frequency for all the subjects were calculated.
3. Processes 1–2 were repeated until Task 14, and the average of all tasks in terms of the percentage of accumulated data/information were calculated for each frequency.
4. The trend of the accumulated information was observed for each frequency, and a frequency having a small increase was selected. To restore up to the corresponding frequency component, the sampling rate was set to twice the frequency component based on the Nyquist sampling theory [30].

The accuracy, training time, and evaluation time of the model were compared before and after the data down-sampling.

##### Data Augmentation Using the Over-Sampling Technique

A medical dataset can easily become unbalanced because it is difficult to obtain negative class data [31]. Therefore, the amount of BBS motion data for each score was unequal. If the model is trained on an unbalanced dataset, the model may be biased toward the majority class, leading to poor performance [32,33]. One way of solving this problem is to balance the dataset by generating new data similar to the original [34,35,36]. Similarly, BBS motion data can be improved using an oversampling technique [37]. As in Khorshidi’s study [38], an over-sampling technique was applied to both the majority and minority classes to equalize the amount of data in each and to increase the total number. Steps 1–3 below describe the data augmentation process:

1. The class set of the scores was $A_{s}$. The number of k samples with the closest Euclidean distance to a random sample, $x\;\left(x\;\epsilon \;A_{n}\right)$, is $x_{k}\;(x_{k}\epsilon \;A_{n})$. $x_{k}$ can be obtained using the k-nearest neighbor algorithm.
2. The number of n (n≤k) new samples between x and $x_{k}$ is $x_{n}$, and the rule for generating $x_{n}$ is given by Equation (1):
  (1) $$x_{n}=x+rand\left(0,1\right)*\left|x-x_{k}\right|$$
3. Steps 1 and 2 are repeated, so that the amount of class data in each class $(A_{0}\sim A_{4}$) becomes N.

To evaluate the model performance, k = 2 and N = 60 were applied using the augmentation process.

#### 2.2.2. Classification Model

In this study, 1D-CNN and GRU ensemble classification models were introduced for the BBS scoring algorithm. The 1D-CNN and LSTM models often show good performance on multivariate time-series data [39,40,41]. Because the amount of BBS data is small, each 1D-CNN and GRU model was constructed with a shallow structure, which is advantageous for small amounts of data [42,43]. The following describes the 1D-CNN and GRU structures used in the experiment and the ensemble model that showed the best performance.

##### 1D-CNN Head and GRU Head

The 1D-CNN head has one convolution layer followed by a max-pooling layer with a size of two, followed by a flattening layer. The kernel size of the convolution layer was three, the number of filters was 64, and the rectified linear unit was used as the activation function. The padding option was the same, and stride was set to one.

The GRU head had a one-time-distributed GRU layer, and its output was flattened. The input size of the GRU unit was 64, and the output size was 64. When using a non-time-distributed GRU layer, the information of all units in the layer was compressed into one vector having a fixed size. Therefore, if the input is long, information may be lost, leading to low model performance [44]. However, the time-distributed GRU layer outputs a feature vector for each unit, and this problem can be alleviated.

##### 1D-CNN, GRU Stacking Ensemble Model

The 1D-CNN and GRU stacking ensemble model is composed of three heads. The three heads include two 1D-CNN heads having a kernel size of one and three, and one GRU head is composed of a one-time distributed GRU layer. The outputs of the three heads are then concatenated, followed by a dense layer with 100 perceptrons. In this case, between these two layers, a 50% dropout was applied to prevent overfitting and to generalize the model. The last layer was a softmax with five perceptrons.

**Figure 5:**
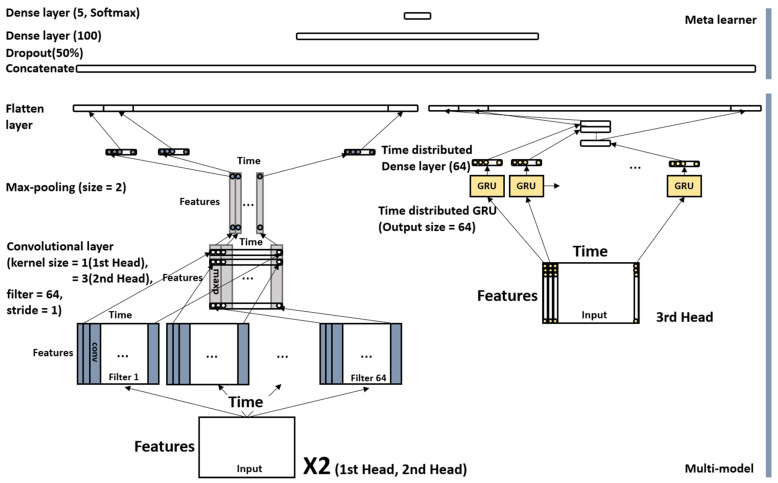
Structure of the 1D-CNN and GRU stacking ensemble model.

The overall structure was a stacking ensemble. The three heads represented each of the models, and the subsequent layers were meta-learners. The meta-learner was equally applied to the proposed model and other experimental models. Figure 5 shows the structure of the 1D-CNN and GRU stacking ensemble model.

##### Training and Evaluation

The model optimizer was Adam with a learning rate of 0.001. The loss function used was the categorical cross entropy, and the batch size was optimized for each task. $Batch\;size_{1\sim 14}=\left\{64,\;32,\;64,\;16,\;32,\;32,\;32,\;64,\;32,\;64,\;64,\;64,\;64,\;64\right\}$. Early stopping was applied; the training was completed when the loss no longer decreased, the patience number was 20, and the maximum number of epochs was limited to 500.

When evaluating the accuracy of a model with data randomly split into training and testing, there may be differences in the accuracy, depending on the split data. Therefore, the model performance was evaluated using the average of the Stratified K-fold cross-validation accuracy. Stratified K-fold cross-validation maintains the ratio of the amount of data per class of the original dataset when splitting the training and test data in K-fold cross-validation. Because the amount of data for each class was equalized by improving the dataset, the data for each class for training and testing were also equalized. Only accuracy was used as the evaluation metric because the model was trained on balanced data; hence, it was not necessary to use an evaluation metric such as the F1 score used in the case of imbalanced data [45]. When evaluating the model, the average performance of all BBS tasks was used; this was to make a good model that could cover all BBS tasks, as in previous studies [3,27].

## 3. Results and Discussion

### 3.1. Improving Model Efficiency through a Data Down-Sampling Process

**Figure 6:**
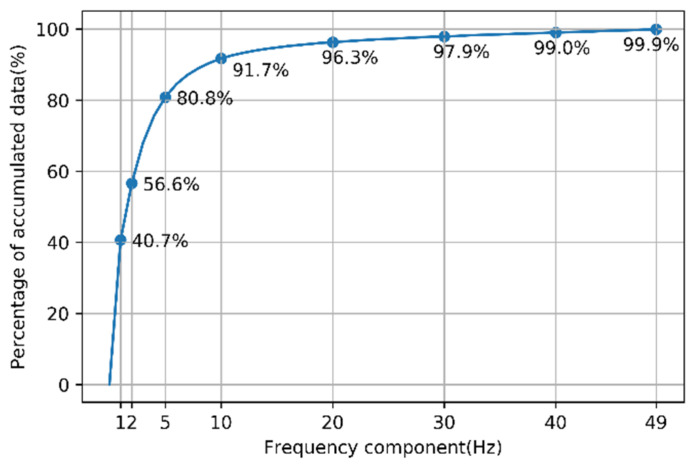
Percentage of accumulated data with respect to the frequency.

To determine an appropriate sampling rate, the accumulated data for each frequency were analyzed. Figure 6 shows the amount of accumulated data with respect to the frequency.

As the frequency increases, the increase in the amount of data tends to decrease. The sum of the frequency components under 10 Hz was more than 90% of the total information, thus confirming that most of the information is in the low-frequency region. In the 5–10 Hz range, the increase in the information rapidly decreases and thereafter remains small. Therefore, the appropriate sampling rate was set such that the frequency component below 10 Hz could be restored. According to the Nyquist sampling theory [30], the sampling rate required to restore a frequency component of n Hz is 2 × n Hz. Therefore, the appropriate sampling rate was set to 20 Hz.

**Figure 7:**
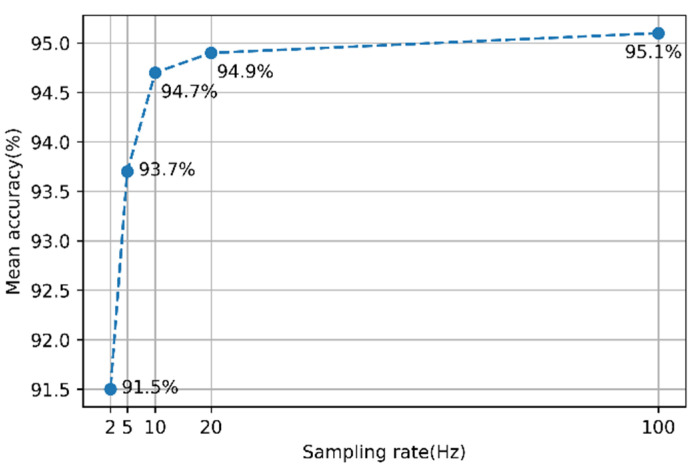
Sampling rate vs. accuracy of the 1D-CNN model.

When down-sampling data from 100 to 20 Hz, the amount of data was reduced by 80%; however, the amount of information was reduced by 8.3%. The classification performance of the scoring model was compared to the data before and after down-sampling to determine the degree to which this loss of information affects the scoring performance. Figure 7 shows the scoring accuracy of 14 tasks using the 1D-CNN model before and after down-sampling. Input data of the model was preprocessed multidimensional time-series data output from the eight IMUs.

When the sampling rate was 20 Hz, the average accuracy of the model decreased by 0.2%. When the data sampling rate was 20 Hz, the training time was reduced by 67.7% compared with when the sampling rate was 100 Hz; in addition, the epoch time was reduced by 66.4%, and the evaluation time was reduced by 58.6%. After down-sampling the data, the gain in the computational efficiency of the model was greater than the loss, owing to the performance decrease caused by information loss. As shown in Figure 7, when the sampling rate was below 20 Hz, the decrease in accuracy was greater; therefore, it was not appropriate to further lower the sampling rate. By observing the graph of Figure 6 and Figure 7, given the similarity in the shapes, the correlation between the amount of information and the model performance is considered to be high. Therefore, predicting the decrease in the model performance based on the amount of information is a reasonable method.

### 3.2. Classification Model

The 1D-CNN and RNN based models, which show good performance on multivariate time-series data, were used as the scoring algorithms. In many studies, the performance could be improved with the 1D-CNN- and RNN-based ensemble models rather than using them alone [23,46,47,48]. This study also tried to find the model structure with the best performance by combining the 1D-CNN model and the RNN-based model using various model structures. Table 2 presents the average performance of the model in the 14 tasks. The performance on each task is the average performance of 10-fold cross-validation. The model names listed in the table are abbreviated as follows: 1D-CNN: C; GRU: G; Double head 1D-CNN: DC; Triple-head 1D-CNN: TC; 1D-CNN after GRU: C-G; 1D-CNN and GRU parallel: C+G; and double-head 1D CNN and GRU parallel: DC+G.

Before constructing the ensemble model, the parameters of the single 1D-CNN and GRU models were optimized, and their performance was checked. Between the two models, the mean accuracy of the GRU model was 95.6%, which is 0.7% higher than that of the 1D-CNN model. However, the training time of the 1D-CNN model was about 76% shorter than that of the GRU model. Therefore, the 1D-CNN and GRU model were both excellent. After this test, various 1D-CNN and GRU ensemble models were tested to find the optimal model.

First, a double-head 1D-CNN model with kernel sizes of one and three was tested for the scoring algorithm. In previous studies [49,50], the performance of multi-head 1D-CNN was found to be better than that of the single-head 1D-CNN and LSTM. The test results showed that the mean accuracy of the double-head 1D-CNN was 95.6%, which is 0.7% higher than that of the 1D-CNN single model and the same as that of the GRU single model. However, the training time was 60% shorter than that of the GRU single model. Therefore, it could be helpful in improving performance. Additionally, triple-head 1D-CNN models with kernel sizes of one, three, and five were tested. The experimental results showed that the performance of the triple-head 1D-CNN was not better than that of the double-head 1D-CNN because the mean accuracy of the triple-head 1D-CNN model was 0.3% lower than that of the double-head 1D-CNN model, and the training time of the triple-head 1D-CNN model was 23% longer than that of the double-head 1D-CNN model; hence, adding three or more 1D-CNN heads did not improve performance.

Second, a model comprising a GRU layer after the 1D-CNN layer was tested. It is natural for the GRU layer to come after the CNN layer in theory [51]. Therefore, many studies have used this model and have obtained good performance [52,53]. The test results showed that the mean accuracy of the 1D-CNN after the GRU model was 95.3%, which is 0.4% higher than that of the 1D-CNN single model, but it was 0.3% lower than that of the GRU single model. Additionally, the training time of the 1D-CNN after the GRU model was 59% longer, and the mean accuracy was 0.3% lower than that of the double-head 1D-CNN model.

Third, the 1D-CNN and GRU parallel models were tested. In XU’s study [54], the CNN and LSTM parallel models outperformed the 1D-CNN and LSTM single models. From the test results, the mean accuracy of the 1D-CNN and GRU parallel models was 95.6%, which is 1.0 and 0.3% higher than that of the 1D-CNN and GRU single models, respectively. It was also 0.3% higher than the double-head 1D-CNN model, whose mean accuracy was the highest.

From the previous test results, the performance improved in two cases: double-head 1D-CNN model and 1D-CNN and GRU parallel models. Therefore, a model with two 1D-CNN heads and one GRU head, which is a stacking ensemble model, was tested. The results of the test showed that the mean accuracy of the two 1D-CNN heads and one GRU head model was 96.1%, which was the highest of all tested models. Additionally, the stability of this model was the best because the standard deviation of the accuracy of the model was 0.2–0.9% lower than that of the other models.

### 3.3. Improvement in Model Performance through Data Augmentation

**Figure 8:**
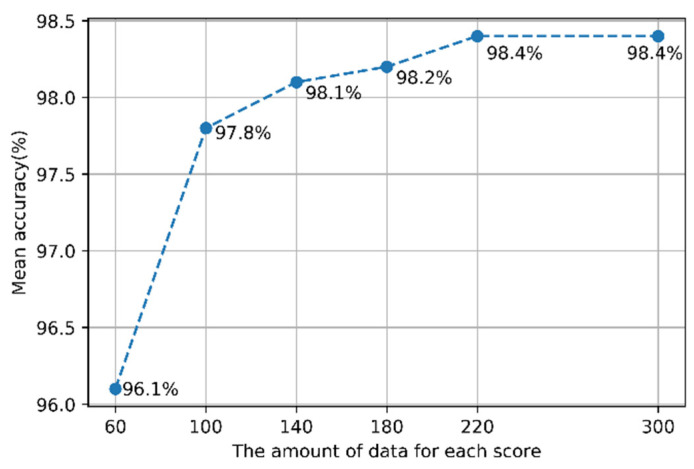
Accuracy of the best model with respect to the amount of data for each score.

One of the objectives of this study was to develop a model that shows good performance in all BBS tasks. Therefore, the amount of data for each score in all the tasks was made the same so that the effect of augmentation was equal for each task. The amount of data for each score was set to 60 for the model tests. Because Task 2 “Standing unsupported,” which had the most imbalanced data, had 56 participants’ motion data with Score 4. The under-sampling technique was not considered because over-sampling showed generally better performance in the data imbalance problem [37]. Many studies have improved the performance of classification models using the oversampling technique [34,35,36,55]. However, the over-sampling technique also decreases the model performance because new data increases noise or can cause overlapping between classes [56]. Therefore, the amount of data for each score was fixed at 60 to reduce the complexity of the experiment. Subsequently, using the model with the best performance, the test was performed to determine whether the model performance could be improved when the amount of data was increased. Figure 8 shows the model performance with respect to the amount of data for each score.

**Figure 9:**
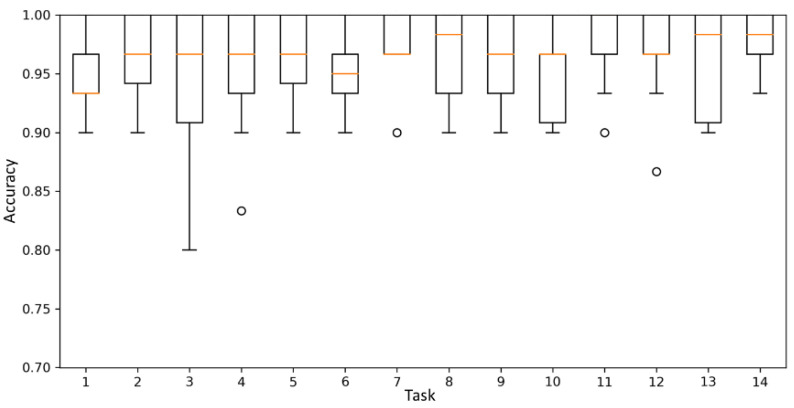
Mean accuracy of the best model when the amount of data for each score was 60.

**Figure 10:**
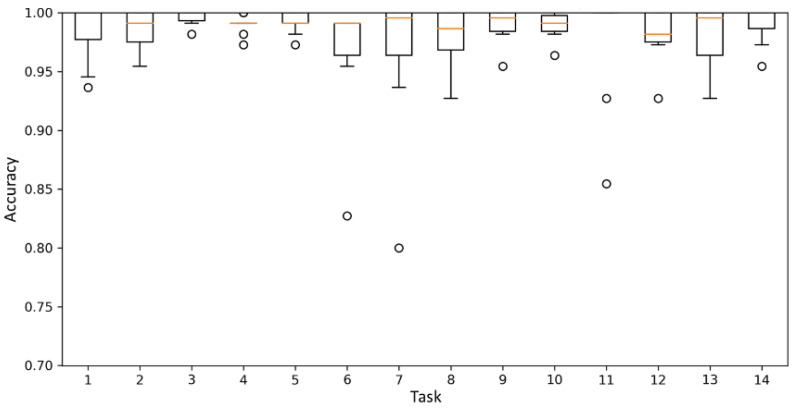
Mean accuracy of the best model when the amount of data for each score was 220.

The average accuracy increases with the increase in the amount of data for each score. The model performance was saturated when the amount of data for each score was 220. Therefore, it was confirmed that the appropriate amount of data for each score to maximize the model performance was 220. When the amount of data for each score was 220, the average accuracy on the 14 tasks was 98.4%. Figure 9 and Figure 10 show boxplots of the accuracy of the model in the 14 tasks when the amount of data for each score was 60 and 220, respectively. The accuracy increased, and the variance of the accuracy decreased when the amount of data for each score was 220.

### 3.4. Comparison with Previous Study

This study evaluated model excellence by comparing the results of previous studies. Prior studies include those of Badura [3] and Kim [27]. Kim’s was a study in which the performance was improved by changing the feature extraction process and machine learning classification model of Badura’s study. Unlike previous studies, this study applied a deep-learning algorithm and performed feature extraction using the deep-learning model. The data sampling rate of the previous studies (100 Hz) was down-sampled to 20 Hz, and the computational complexity was improved without reducing model accuracy. Reducing the sampling rate of data is meaningful in that it can contribute to reducing power consumption by lowering the sampling rate of the wearable devices. The dataset of the previous study had a data imbalance problem in which the amount of data for each score was different. In this study, this problem was solved by performing data augmentation based on the oversampling algorithm. As a result, the classification accuracy was increased. Additionally, a healthy participant experiment was performed to compensate for the insufficient amount of data in some classes. Because healthy participants performed all actions from zero to four on all tasks, the amount of data was equal to five times the patient data per healthy participant. The total amount of data was 78, 53 of which were patient data, and 15 were created by three healthy participants. By using the 10-fold cross-validation average accuracy as the evaluation method of the model, the evaluation method of previous studies, which can produce good results only in some splits, was improved overall. Table 3 summarizes the improvement points of this study compared with these previous studies.

The main achievement of this study is the improvement of accuracy. Table 4 shows the scoring accuracy of the model in the previous studies and the model that showed the best performance in this study. The model names listed in the table are abbreviated as follows: double-head 1D CNN and GRU stacking ensemble: DC+G.

The average accuracy of the model in this study was about 18% higher than that of the multi-layer perceptron model of Badura’s study [3] and about 5% higher than that of the SVM model of Kim’s study [27]. It was also confirmed that the standard deviation of the accuracy for the BBS task from 1 to 14 of the models of this study was 0.7%, which was much smaller than 10.9% of the MLP model of Badura’s study and 9.1% of Kim’s SVM model. This means that the model of this study can cover all BBS tasks well.

## 4. Conclusions

In this study, a deep learning-based BBS auto-scoring algorithm was developed. The best model was the stacking ensemble model with a meta-learner comprising a simple dense layer behind two 1D-CNN heads and one GRU head. The computational complexity and accuracy of the model were improved by improving the dataset during preprocessing. During the down-sampling process, it was possible to find a reasonable sampling rate by analyzing the accumulated information amount and the trend in the accumulated information amount with respect to the frequency change. After down-sampling, the computational complexity of the model was reduced. During the data augmentation process, the dataset was improved using the over-sampling technique. By creating data similar to the original data, the amount of data for each score was equalized, and that of both minority and majority classes was increased so that the deep-learning model could learn the data more generally. As a result, the scoring performance of the model was improved without a performance decrease caused by noise or class overlapping that occurs otherwise due to the generated data [56]. The accuracy was saturated when the amount of data for each score exceeded a certain threshold. The maximum average accuracy of the model in the 14 tasks was 98.4%, which was superior to previously reported results.

In the previous study [27], the efficiency of the algorithm could be increased by using only sensor data, which is advantageous to score classification for each BBS task. Of course, the deep-learning method performs this process inside the model by adjusting the weights between perceptrons. However, it has a disadvantage in that the amount of computations is large because all sensor data must be entered as an input. Therefore, in a follow-up study, an attention model deep-learning method will be introduced, and weights will be visualized to exclude data having a low contribution to scoring classification. This will not only increase the computational efficiency of the model, but it will also have the advantage that users can wear fewer sensors. Furthermore, we will introduce the latest deep-learning techniques and improve the dataset with signal processing algorithms to increase performance.

The algorithm of this study can be applied to a wearable healthcare device that evaluates the balance ability in the daily life of elderly or brain-disease patients who are at risk of falling. With wearable healthcare devices, users can know their balance ability and probability of falling at any time without having to visit a hospital, and it will be helpful for falling prevention.

## Figures and Tables

**Table 1:** Berg balance scale tasks.

No.	Task Description
1	Sitting to standing
2	Standing unsupported
3	Sitting unsupported
4	Standing to sitting
5	Transfers
6	Standing with eyes closed
7	Standing with feet together
8	Reaching forward with outstretched arms
9	Retrieving object from floor
10	Turning to look behind
11	Turning 360°
12	Placing alternate foot on stool
13	Standing with one foot in front
14	Standing on one foot

**Table 2:** Performance of 1D-CNN, GRU-based model.

Model	C	G	DC	TC	C-G	C+G	DC+G
Mean accuracy (%)	94.9	95.6	95.6	95.3	95.3	95.9	96.1
Standard deviation of accuracy (%)	4.4	4.1	4.0	4.4	4.7	4.1	3.8
Max accuracy (%)	99.8	99.8	100	99.8	100	100	100
Min accuracy (%)	87.1	87.4	87.6	86.4	85.7	87.2	88.8
Mean epoch	64.9	80.6	69.9	63.8	80.1	71.7	78.8
Mean training time (s)	5.172	21.351	8.383	10.270	13.304	21.729	26.551
Epoch time (s)	0.081	0.265	0.120	0.161	0.166	0.303	0.337
Evaluation time (s)	0.099	0.073	0.142	0.153	0.115	0.095	0.129

**Table 3:** Improvements in this study compared to previous studies.

Study	Badura’s Study	Kim’s Study	This Study
Classification model	Multi-layer perceptron (MLP)	Support vector Machine (SVM)	Double head 1D-CNN and single head GRU stacking ensemble
Feature extraction	Manual (Frequency and time domain feature, Feature selection: Fisher’s linear discriminant)	Manual (Frequency domain and energy feature, Feature selection: KPCA)	Automatic in deep learning
Sampling rate of data (Hz)	100	100	20 (Introduce data down-sampling)
Data imbalance problem	Yes	Yes	No (Introduce data augmentation)
Amount of experimental data	63	53	78
Evaluation method	Random split Training: Test = 7:3	Random split Training: Test = 7:3	Mean accuracy of 10-fold cross validation

**Table 4:** Comparison of results of previous study with this study.

Task	Badura’s MLP Accuracy (%)	Kim’s SVM Accuracy (%)	DC+G Accuracy (%)
1	87.5	100	98.5
2	92.2	100	98.5
3	100	100	99.6
4	89.1	87.5	99.0
5	70.3	76.5	96.7
6	89.1	100	97.9
7	76.6	100	99.0
8	76.6	92.9	98.9
9	89.1	100	97.8
10	70.3	78.6	98.2
11	78.1	100	97.8
12	79.7	80.0	98.2
13	62.5	90.0	98.1
14	67.2	100	99.1
Average	80.6	93.2	98.4
Standard deviation	10.9	9.1	0.7
